# Aestivation Induces Changes in the mRNA Expression Levels and Protein Abundance of Two Isoforms of Urea Transporters in the Gills of the African Lungfish, *Protopterus annectens*

**DOI:** 10.3389/fphys.2017.00071

**Published:** 2017-02-16

**Authors:** You R. Chng, Jasmine L. Y. Ong, Biyun Ching, Xiu L. Chen, Kum C. Hiong, Wai P. Wong, Shit F. Chew, Siew H. Lam, Yuen K. Ip

**Affiliations:** ^1^Department of Biological Sciences, National University of SingaporeSingapore, Singapore; ^2^Natural Sciences and Science Education, National Institute of Education, Nanyang Technological UniversitySingapore, Singapore; ^3^NUS Environmental Research Institute, National University of SingaporeSingapore, Singapore

**Keywords:** ammonia, nitrogenous waste, urea excretion, Ut-a2a, Ut-a2b

## Abstract

The African lungfish, *Protopterus annectens*, is ammonotelic in water despite being ureogenic. When it aestivates in mucus cocoon on land, ammonia is detoxified to urea. During the maintenance phase of aestivation, urea accumulates in the body, which is subsequently excreted upon arousal. Urea excretion involves urea transporters (UT/Ut). This study aimed to clone and sequence the *ut* isoforms from the gills of *P. annectens*, and to test the hypothesis that the mRNA and/or protein expression levels of *ut*/Ut isoforms could vary in the gills of *P. annectens* during the induction, maintenance, and arousal phases of aestivation. Two isoforms of *ut, ut-a2a* and *ut-a2b*, were obtained from the gills of *P. annectens*. *ut-a2a* consisted of 1227 bp and coded for 408 amino acids with an estimated molecular mass of 44.7 kDa, while *ut-a2b* consisted of 1392 bp and coded for 464 amino acids with an estimated molecular mass of 51.2 kDa. Ut-a2a and Ut-a2b of *P. annectens* had a closer phylogenetic relationship with Ut/UT of tetrapods than Ut of fishes. While the mRNA expression pattern of *ut-a2a* and *ut-a2b* across various tissues of *P. annectens* differed, the transcript levels of *ut-a2a* and *ut-a2b* in the gills were comparable, indicating that they might be equally important for branchial urea excretion during the initial arousal phase of aestivation. During the maintenance phase of aestivation, the transcript level of *ut-a2a* increased significantly, but the protein abundance of Ut-a2a remained unchanged in the gills of *P. annectens*. This could be an adaptive feature to prepare for an increase in the production of Ut-a2a upon arousal. Indeed, arousal led to a significant increase in the branchial Ut-a2a protein abundance. Although the transcript level of *ut-a2b* remained unchanged, there were significant increases in the protein abundance of Ut-a2b in the gills of *P. annectens* throughout the three phases of aestivation. The increase in the protein abundance of Ut-a2b during the maintenance phase could also be an adaptive feature to prepare for efficient urea excretion when water becomes available.

## Introduction

Lungfishes are an archaic group of Sarcopterygian fishes that possess lung for air-breathing. At present, there are six extant lungfish species in the world, of which four species can be found in Africa, namely *Protopterus aethiopicus, P. amphibicus, P. annectens*, and *P. dolloi*. During drought, African lungfishes can aestivate in subterranean mud cocoons for as long as 4 years (Ballantyne and Frick, 2010; Ip and Chew, 2010a; Chew et al., 2015). Aestivation refers to a state of corporal torpor adopted by some animals to survive arid and torrid conditions without water or food intake for an extended period. African lungfishes can be induced to aestivate in dried mucus cocoon in plastic tanks in the laboratory (Chew et al., 2004; Ip et al., 2005a; Loong et al., 2005).

Aestivation comprises three phases: induction, maintenance, and arousal (Chew et al., 2015). During the induction phase of aestivation, the aestivating lungfish detects cues from the environment and turns them into internal signals that result in behavioral, physiological, structural, and biochemical changes to prepare for the maintenance phase. In the first 6–8 days, it hyperventilates and secretes mucus which turns into a dry mucus cocoon. During the maintenance phase of aestivation, the lungfish is fully encased in a cocoon and all locomotor and feeding activities cease. The lungfish can perpetuate to aestivate in these conditions in the laboratory for more than a year, during which it has to preserve biological structures, avoid cell death, and sustain a slow rate of waste production to prevent pollution of the internal environment. The lungfish can be aroused from aestivation upon the addition of water. It has to excrete the accumulated waste products and feed for growth and repair during the arousal phase of aestivation. Approximately 7–10 days after arousal, feeding begins and the fish would grow and develop as normal. While metabolic changes are expected to vary between the three phases of aestivation, most reports in the literature focus mainly on the maintenance phase.

In water, African lungfishes are ammonotelic (Chew et al., 2003), and it has been established that fish gills constitute the main organ for ammonia excretion (Wilkie, 1997, 2002; Weihrauch et al., 2009). However, without water to flush the gills, ammonia excretion would be impeded during aestivation on land. As ammonia is toxic (Chew et al., 2006; Ip and Chew, 2010b; Chew and Ip, 2014), African lungfishes defend against ammonia toxicity through increased urea synthesis and decreased ammonia production during aestivation (Chew et al., 2004; Ip et al., 2005a; Loong et al., 2005). During the induction phase, the mRNA expression levels of *carbamoyl phosphate synthetase III* (*cps III*; Loong et al., 2012), *argininosuccinate synthase* (*ass*) and *argininosuccinate lyase* (*asl*; Chng et al., 2014) increase significantly in the liver of *P. annectens*, indicating an increase in urea synthesis. Urea cannot be excreted by the aestivating lungfish, and the urea accumulated in the body may function as a putative internal cue for aestivation (Ip et al., 2005b). Upon arousal, African lungfishes can efficiently excrete urea accumulated during the maintenance phase (Ip and Chew, 2010a). For *P. dolloi* exposed to terrestrial conditions for 6 days, the rate of urea excretion increased by 22-fold during re-immersion as compared to the control fish (Chew et al., 2003). This is the greatest increase in urea excretion reported for fishes during emersion-immersion transition, and implies that there are transporters which facilitate urea excretion upon arousal in *P. dolloi* (Chew et al., 2003).

Urea transporters (UT) are members of the solute transporter family SLC14 that facilitate the passive diffusion of urea down its concentration gradient (Levin et al., 2012). In mammals, UT-A, which comprises six isoforms, and UT-B, which comprises two isoforms, are derived from two distinct genes, *SLC14A2* and *SLC14A1*, respectively (Bagnasco, 2003; Smith, 2009; Stewart, 2011). They are expressed in a wide variety of tissues (Sands and Blount, 2014). However, the function of UT is best understood in the kidney where they contribute to maintaining high interstitial urea concentration required to limit the rate of water loss (Hediger et al., 1996; Sands, 2003; Klein et al., 2011). Hung et al. (2009) obtained the complete coding sequence of only one putative urea transporter (*lfut*) from the skin of *P. annectens*, which was also expressed in the gills, kidney, liver and skeletal muscle. As several gene duplication events of *ut* have occurred during the evolution of lower vertebrates, LeMoine and Walsh (2015) postulated that the presence of a single *ut* in lungfishes implied the loss of duplicated *ut* isoforms prior to the Sarcopterygian radiation. Furthermore, Hung et al. (2009) reported that, upon re-immersion in water after 33 days of air exposure, there were increases in urea excretion in *P. annectens*, which peaked at 12–30 h and lasted until hour 70. The mRNA expression level of *lfut* increased significantly in the skin 14–48 h after re-immersion, denoting the skin as the site of increased urea excretion in the aroused lungfish. Notably, lungfish specimens were exposed to “terrestrialization” before re-immersion in the study of Hung et al. (2009). “Terrestrialization” was a term used originally by Wood et al. (2005) and Wilkie et al. (2007) to describe an extended period of aerial exposure in *P. dolloi* with the occasional addition of water to prevent the complete formation of a dried cocoon (see Chew et al., 2015 for a review). Unlike aestivating lungfishes, those undergoing terrestrialization were not confronted with desiccation and displayed occasional movement (Chew et al., 2015). In essence, fish exposed to terrestrialization were sustained in a prolonged induction phase of aestivation. As such, there is no information on the expression of *ut*/Ut in the gills of African lungfishes during the maintenance phase.

Therefore, this study was undertaken to clone and sequence the cDNA coding region of *ut* isoforms from the gills of *P. annectens*. It was hoped that the deduced Ut amino acid sequences would provide insights on the phylogenetic relationship between *P. annectens* and other animals. Moreover, quantitative real-time PCR (qPCR) was conducted to determine the branchial mRNA expression levels of *ut* isoforms in *P. annectens* kept in fresh water (control) or aestivated for 6 days (the induction phase of aestivation) or 6 months (the maintenance phase of aestivation), or aroused for 1 day or 3 days from 6 months of aestivation (the arousal phase of aestivation). Western blotting was conducted to determine the branchial protein abundance of Ut isoforms, using custom-made antibodies developed based on the deduced Ut sequences. It was expected that results obtained would provide insights on whether the mRNA and protein expression of *ut*/Ut in the gills of *P. annectens* would be down-regulated during the induction and maintenance phases of aestivation to impede urea excretion and/or to shut down branchial functions, and whether they would be up-regulated during the initial arousal phase to facilitate the excretion of the urea accumulated in the body.

## Materials and methods

### Animals

Specimens of *P. annectens* (80–150 g body mass) were imported from Central Africa through a fish farm in Singapore. In the laboratory, they were kept in plastic tanks filled with dechlorinated tap water at 25°C. There was no attempt to separate the sexes. Water was changed daily. Fish were acclimated to laboratory conditions for at least 2 weeks and fed with frozen fish meat during the acclimatization period. The procedures used in this study have been approved by the Institutional Animal Care and Use Committee of the National University of Singapore (IACUC 035/09).

### Experimental conditions and sample collection

Following the procedure of Chew et al. (2004), *P. annectens* were induced to aestivate individually in plastic tanks (85–90% humidity and 27–29°C) containing 15 ml dechlorinated tap water (made Salinity = 0.3 with seawater). It took around 6 days for the lungfish to be encased in a brown dried mucus cocoon. In this study, these 6 days were counted as part of the aestivation period and regarded as the induction phase of aestivation. The lungfish were allowed to aestivate for 6 months. After 6 months of aestivation, some lungfish were aroused by adding 200 ml of water into the tank. Subsequently, the aroused lungfish was covered with an additional 800 ml of water.

Lungfish (*N* = 4) kept in fresh water acted as the control and they were killed with an overdose of neutralized 0.05% MS222 after withholding food for 96 h. After 6 days or 6 months of aestivation, some lungfish (*N* = 4 for each group) were pithed after a blow to the head. After 1 day or 3 days of arousal from 6 months of aestivation, some lungfish (*N* = 4 for each group) were killed with an overdose of neutralized 0.05% MS222. The gills, eye, brain, heart, liver, spleen, pancreas, gut, kidney, lung, muscle and skin were quickly excised and freeze-clamped with aluminum tongs pre-cooled in liquid nitrogen.

### Total RNA extraction and cDNA synthesis

Total RNA extraction was performed on samples of *P. annectens* using Tri Reagent™ (Sigma-Aldrich Co., St. Louis, MO, USA). RNA was purified by Qiagen RNeasy Mini Kit (Qiagen GmbH, Hilden, Germany) and quantified by BioSpec-nano (Shimadzu, Tokyo, Japan). The integrity of RNA was assessed electrophoretically and gauged by the ratio of 28S/18S rRNA. The purity of RNA was accessed by the absorbance ratio of A260/A280. First strand cDNA synthesis was performed using 4 μg of total RNA, oligo(dT)_18_ primer and the RevertAid™ first strand cDNA synthesis kit (Thermo Fisher Scientific, Waltham, MA, USA).

### Polymerase chain reaction (PCR), molecular cloning, and rapid amplification of cDNA ends (RACE)

In order to obtain the partial sequences of *ut-a2a* and *ut-a2b* from the gills of *P. annectens*, gene-specific PCR primers (Table 1) were designed from highly conserved regions of related sequences from several fish species available in Genbank. Furthermore, efforts were made to verify whether *ut-a2a* obtained from the gills of *P. annectens* in this study was identical to the *lfut* sequence (accession: EU716115.1) obtained from the skin by Hung et al. (2009). PCR was performed using Dreamtaq polymerase (Thermo Fisher Scientific) with thermal cycling conditions: 95°C (3 min), accompanied by 40 cycles of 95°C (30 s), 60°C (30 s), 72°C (2 min) and a final extension of 72°C (10 min). Gel electrophoresis was performed to separate the PCR products, and the band of estimated molecular mass was excised. FavorPrep™ Gel Purification Mini Kit (Favorgen Biotech Corp., Ping Tung, Taiwan) was used to purify the excised PCR product. Sequencing was conducted by a 3130XL Genetic Analyzer (Life Technologies Corporation, Carlsbad, California), using BigDye® Terminator v3.1 Cycle Sequencing Kit (Life Technologies Corporation).

**Table 1:** **Primers used for PCR, RACE, and qPCR of *urea transporter a2a* (*ut-a2a*) and *urea transporter a2b* (*ut-a2b*) from the gills of *Protopterus annectens***.

**Gene**	**Primer type**	**Primer sequence (5′to 3′)**
*ut-a2a*	PCR	Forward: AGGAACTGAAGTCACTCATGG
		Reverse: GGAAGACTGCAATGATATTGTG
	5′-RACE	TTAGCTTCTGGGTATGTGACTT
	3′-RACE	GGAGACATGGAAGAATTTGG
	qPCR	Forward: AAAGAATCTGTGAAGCCAGTAG
		Reverse: ATCACTTGTGCTGTACCTCTTA
*ut-a2b*	PCR	Forward: AACAGTTCGTAAAGTCATAGCC
		Reverse: CACAGAATCATTGTTGTCTTCC
	5′-RACE	CCACTGCACTTGTCAGAATAGGG
	3′-RACE	CAACTCTGATTCAGCCGCAGACCT
	qPCR	Forward: CTAACACATTTCCGATGACAG
		Reverse: CCTCCTCAGTAGATTGTTCAG

Ligation of the purified PCR products was performed using pGEM®-T Easy vector (Promega Corporation, Madison, WI, USA). Subsequently, the ligation mixtures were transformed into JM109 *Escherichia coli* competent cells. Standard blue/white screening was carried out on LB/ampicillin/bromo-chromo-iodolyl-galactopyranoside (X-gal)/isopropyl β-D-1-thiogalactopyranoside (IPTG) plates. Colony-PCR was then performed on all the selected white colonies. The colonies with insert of expected sizes were selected and grown overnight in LB/ampicillin broth in a shaking incubator (37°C, 250 rpm). The plasmids were extracted using AxyPrep Plasmid Miniprep Kit (Axygen Biosciences, Union City, CA, USA) and sequenced. The partial sequences obtained were verified to be *ut-a2a* and *ut-a2b*.

Using SMARTer™ RACE cDNA Amplification kit (Clontech Laboratories, Mountain View, CA, USA), 1 μg of total RNA from *P. annectens* gills was reverse transcribed into 3′-RACE-Ready cDNA and 5′-RACE-Ready cDNA. Using gene-specific RACE primers (Table 1) and Advantage® 2 PCR kit (Clontech Laboratories), RACE-PCR were conducted to generate the 3′ and 5′ cDNA fragments of *ut-a2a* and *ut-a2b*. The cycling conditions involved 30 cycles of 94°C (30 s), 65°C (30 s), and 72°C (4 min). RACE-PCR products were separated by gel electrophoresis, purified and sequenced. To obtain the full coding sequences of *ut-a2a* and *ut-a2b*, multiple sequencing was conducted in both directions. Bioedit v7.1.3 (Hall, 1999) was used for the assembly and analysis of sequences. The complete coding cDNA sequences of *ut-a2a* and *ut-a2b* have been deposited into Genbank with the accession numbers KX610111 and KX610112, respectively.

### Molecular characterization of the deduced amino acid sequences of Ut-a2a and Ut-a2b

The deduced amino acid sequences of Ut-a2a and Ut-a2b were translated from the nucleotide sequences of *ut-a2a* and *ut-a2b* using ExPASy Proteomic server (http://web.expasy.org/translate/). The alignment of the deduced amino acid sequences with selected Ut/UT from several animal species was performed using Bioedit. Potential *N*-glycosylation sites were verified using NetNGlyc 1.0 and potential phosphorylation sites were verified using NetPhos 2.0. The transmembrane domains of Ut-a2a and Ut-a2b were identified using MEMSAT3 and MEMSAT-SVM in PSIPRED protein structure prediction server (http://bioinf.cs.ucl.ac.uk/psipred/; McGuffin et al., 2000).

### Dendrogramic analysis

The sequences of Ut-a2a and Ut-a2b were aligned using ClustalX2. Dendrogramic analysis was conducted by neighbor-joining method with 100 bootstrap replicates using Phylip v3.6 (Felsenstein, 2001). The accession numbers of selected Ut/UT amino acid sequences used in the dendrogramic analysis are indicated in Table S1.

### mRNA expression in various tissues/organs

Using gene-specific qPCR primers of *ut-a2a* and *ut-a2b* (Table 1), PCR was conducted on the cDNAs of gills, eye, brain, heart, liver, spleen, pancreas, gut, kidney, lung, muscle, and skin of *P. annectens*. Each PCR was performed in 10 μl reaction vols. with cycling conditions: 95°C (3 min), accompanied by 32 cycles of 95°C (30 s), 55°C (30 s), 72°C (30 s) and a final extension of 72°C (10 min). The PCR products were separated by gel electrophoresis.

### qPCR

In this study, the method of absolute quantification with reference to a standard curve was used because it was crucial to compare the transcript levels of *ut-a2a* and *ut-a2b* in *P. annectens* gills. The method of relative quantitation does not allow interpretation of the predominant gene expressed in a certain condition and provides only fold-change data.

The Qiagen RNeasy Plus Mini Kit (Qiagen GmbH), which contains gDNA Eliminator spin column (Qiagen GmbH) to remove genomic DNA, was used to purify the RNA from the gills of *P. annectens*. First strand cDNA synthesis was performed using 4 μg of total RNA, random hexamer primers and RevertAid™ first strand cDNA synthesis kit (Thermo Fisher Scientific). Following the method of Gerwick et al. (2007), a pure amplicon (standard) that was defined by the gene-specific qPCR primers (Table 1) was obtained. PCR was performed with cycling conditions: 95°C (3 min), accompanied by 40 cycles of 95°C (30 s), 60°C (30 s), and 72°C (30 s), and a final extension of 72°C (10 min). Subsequently, the PCR product was separated by gel electrophoresis, excised, and purified. The purified PCR product was cloned using pGEM®-T Easy vector (Promega Corporation). The presence of the insert in the cloned plasmid was validated by sequencing. The selected cloned plasmid that was used as the standard was serially diluted (10^6^–10^2^ specific copies per 2 μl). The standard was used as a reference to determine the absolute quantities of *ut-a2a* or *ut-a2b* transcripts in the qPCR reaction.

qPCR was carried out in duplicates using the StepOnePlus™ Real-Time PCR System (Life Technologies Corporation). Each qPCR reaction contained 0.3 μmol l^−1^ of forward and reverse gene-specific qPCR primers each (Table 1), 5 μl of KAPA SYBR® FAST Master Mix (2X) ABI Prism™ (Kapa Biosystems, Woburn, MA, USA) and various quantities of standard or 1 ng of sample cDNA in a total volume of 10 μl. The cycling conditions were 95°C (20 s), accompanied by 40 cycles of 95°C (3 s), and 60°C (30 s). At each elongation step, the values of the threshold cycle (C_t_) were collected. A melt curve analysis was conducted to verify the presence of a single product. Furthermore, the PCR product was separated by gel electrophoresis to confirm the presence of a single band. A standard curve was produced by plotting the natural log of concentration (copies μl^−1^) on the X-axis and C_t_ on the Y-axis. The default setting of StepOne™ Software v2.1 (Life Technologies Corporation) was used for the calculation of the Y-intercept, C_t_ slope, correlation coefficient (*r*^2^), and PCR efficiency. The PCR efficiencies for the gene-specific primers of *ut-a2a*, and *ut-a2b* were 91.5 and 89.3%, respectively. The linear regression line derived from the standard curve was used to determine the quantity of transcript in an unknown sample, which was expressed as copies of transcripts per ng total RNA.

### Western blotting

Western blotting was conducted on the gills of *P. annectens* kept in fresh water (control), or aestivated for 6 days or 6 months, or aroused for 1 day or 3 days from 6 months of aestivation. Each sample was homogenized twice in five volumes (w/v) of ice cold buffer containing 1× HALT protease inhibitor cocktail (Thermo Fisher Scientific), 150 mmol l^−1^ NaCl, 1 mmol l^−1^ Na_3_VO_4_, 1 mmol l^−1^ NaF, 50 mmol l^−1^ Tris HCl, (pH 7.4), 1 mmol l^−1^ EDTA, 1 mmol l^−1^ phenylmethylsulfonyl fluoride, 1% sodium deoxycholate, and 1% NP-40, using pre-cooled TissueLyser LT (Qiagen GmbH) at 50 Hz for 2.5 min. Subsequently, the homogenate was centrifuged at 10,000 × g for 20 min at 4°C. The protein concentration in the supernatant was determined (Bradford, 1976) and adjusted to 10 μg μl^−1^ with Laemmli buffer (Laemmli, 1970). The samples were then heated at 70°C for 15 min, and kept at −80°C until analysis.

Two rabbit polyclonal antibodies against aa 23–36 (HNTEKQNDKKESVKC) of Ut-a2a and aa 40–53 (LSENTETSVPEYHPC) of Ut-a2b from *P. annectens* were custom-made through a commercial company (GenScript, Piscataway, NJ, USA). The concentration of the anti-Ut-a2a and anti-Ut-a2b antibodies used for Western blotting was 1.7 μg ml^−1^.

Following the method of Laemmli (1970), proteins (200 μg) were separated by SDS-PAGE using a vertical mini-slab apparatus (Bio-Rad Laboratories, Hercules, CA, USA). Using a transfer apparatus (Bio-Rad Laboratories), proteins were electrophoretically transferred onto PVDF membranes. In order to verify the specificity of the anti-Ut-a2a and anti-Ut-a2b antibodies, peptide competition assays were also performed. The anti-Ut-a2a and anti-Ut-a2b (16.7 μg each) antibodies were pre-incubated with their corresponding immunizing peptide (83.5 μg each; Genscript) at 25°C for 1 h and subsequently used for the peptide competition assay. Bands were detected by Pierce™ SuperSignal™ West Pico Rabbit Fast Western Kit (Thermo Fisher Scientific) and visualized by chemiluminescence (Western Lightning™, PerkinElmer Life Sciences, Boston, MA, USA). A Kodak X-Omat 3000 RA processor (Kodak, Rochester, NY, USA) was used to process the X-ray film (Thermo Fisher Scientific). ImageJ (version 1.40, NIH) was used for the densitometric quantification of the band intensities. We were unable to find a reference protein with protein expression that was unaffected throughout the three phases of aestivation. Therefore, the protein abundance of Ut-a2a or Ut-a2b were presented as arbitrary densitometric unit per μg protein.

### Statistical analyses

Results were presented as means ± standard errors of the mean (*S.E.M*.). SPSS version 18 (SPSS Inc, Chicago, USA) was used for statistical analyses. Levene's Test was used to verify the homogeneity of variance. Differences between means were verified using one-way analysis of variance, followed by multiple comparisons of means using either Dunnett T3 or Tukey *post-hoc* test, depending on the homogeneity of variance of the data set. Differences were reported as statistically significant at *P* < 0.05.

## Results

### Nucleotide sequences, deduced amino acid sequences, and dendrogramic analysis

The complete coding cDNA sequence of *ut-a2a* obtained from the gills of *P. annectens* comprised 1227 bp, coding for 408 amino acids with an estimated molecular mass of 44.7 kDa (accession: KX610111). In comparison, the complete coding cDNA sequence of *ut-a2b* comprised 1392 bp, coding for 463 amino acids with an estimated molecular mass of 51.2 kDa (accession: KX610112). The similarity between *ut-a2a* and *lfut* (accession: EU716115.1) was 99.7%, while the similarity between *ut-a2b* and *lfut* was 71.3%.

**Figure 1:**
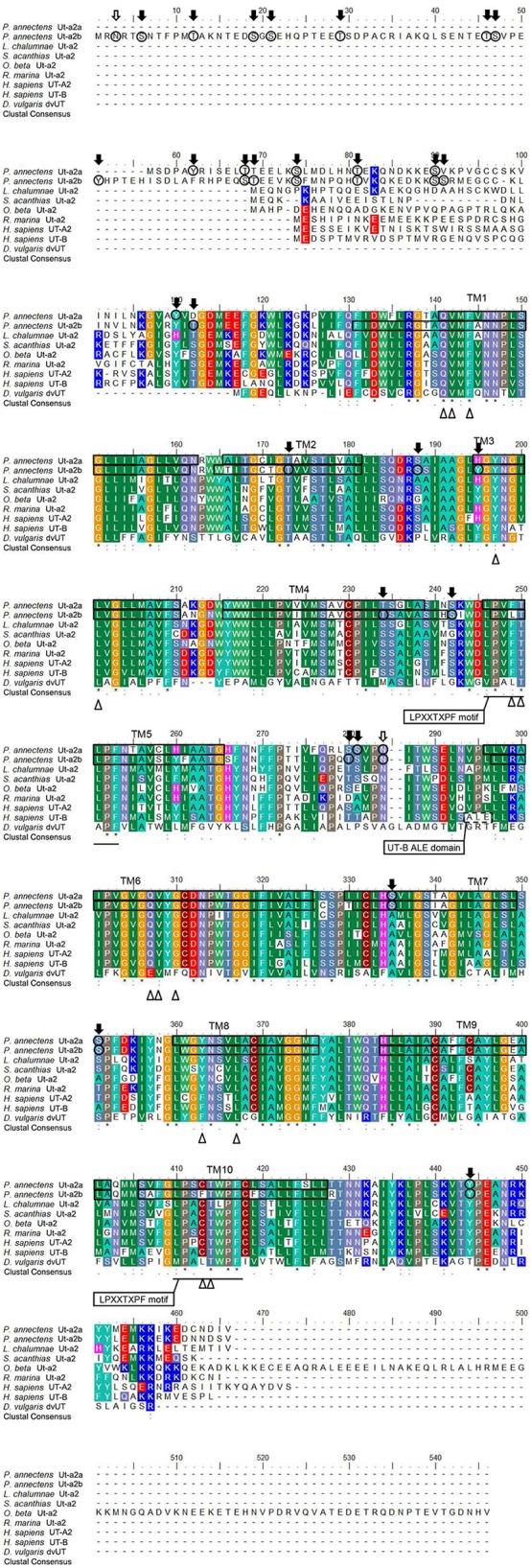
**A multiple amino acid alignment of the isoforms of urea transporter (Ut-a2a and Ut-a2b) from *Protopterus annectens* with *Latimeria chalumnae* Ut-a2 (XP_006007026.1), *Squalus acanthias* Ut-a2 (AAF66072.1)**, ***Opsanus beta*** **Ut-a2 (AAD53268.2)**, ***Rhinella marina*** **Ut-a2 (BAE16706.1)**, ***Homo sapiens*** **UT-A2 (CAA65657.1) and UT-B1 (CAB60834.1), and *Desulfovibrio vulgaris* Ut (Q72CX3)**. Identical amino acid residues are indicated by asterisks, strongly similar amino acids are indicated by colons and weakly similar amino acids are indicated by periods. The conserved LPXXTXPF motifs are underlined. The potential urea binding sites are indicated by open triangles. The dotted box denotes the UT-B signature ALE domain. Potential *N*-glycosylation and phosphorylation sites are indicated by open and shaded arrows, respectively. The predicted transmembrane domains (TM1-10) of Ut-a2a and Ut-a2b of *P. annectens* are indicated by open boxes and were predicted using MEMSATS and MEMSAT-SVA provided by PSIPRED protein structure prediction server.

**Figure 2:**
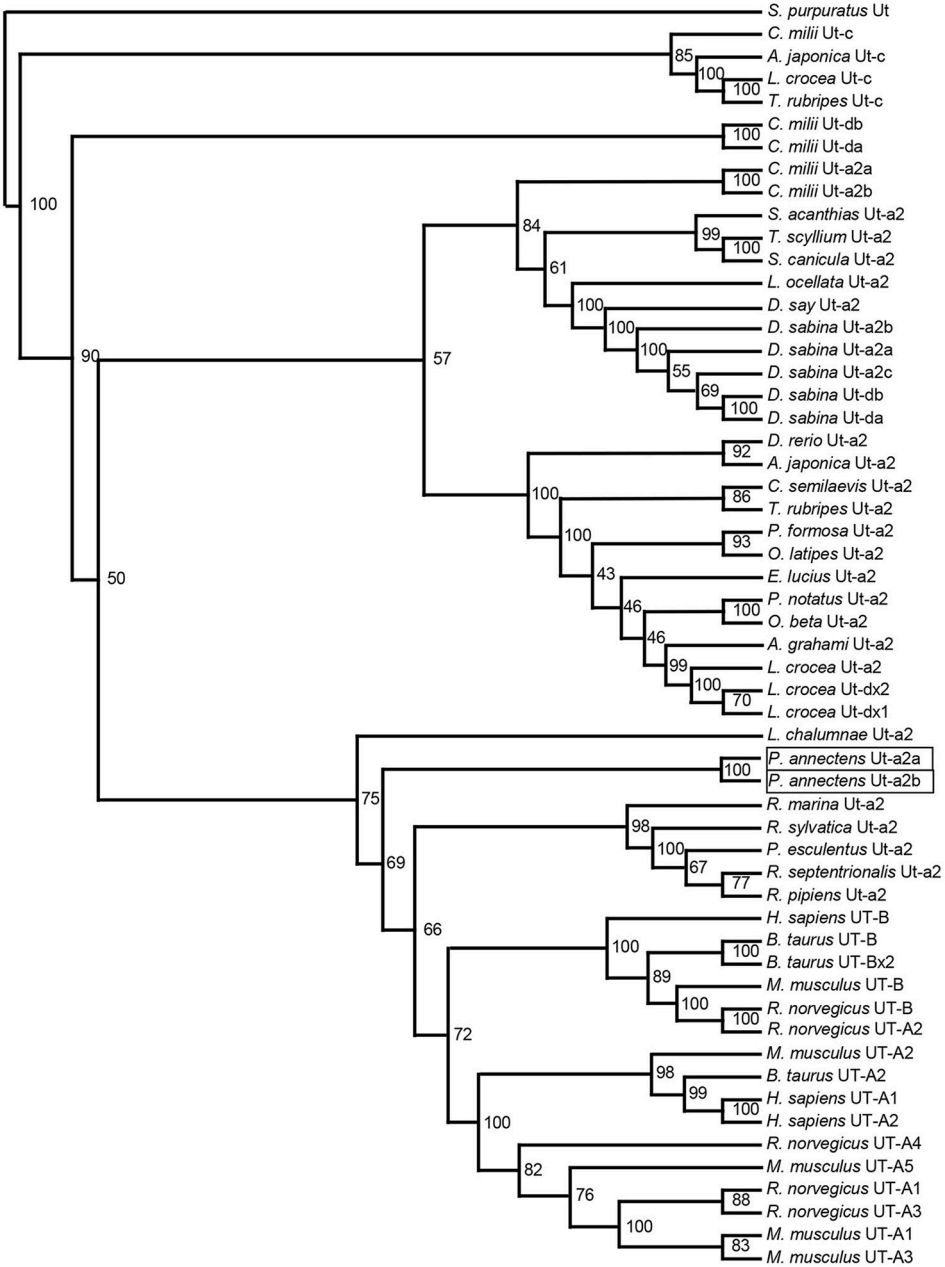
**A dendrogram of urea transporters (Ut/UT) including Ut-a2a and Ut-a2b of *Protopterus annectens***. *Strongylocentrotus purpuratus* Ut is used as outgroup for the dendrogram. Numbers presented at each branch point represent bootstrap values from 100 replicates.

A hydropathy analysis indicated that the deduced amino acid sequences of Ut-a2a and Ut-a2b of *P. annectens* comprised 10 transmembrane regions. Efforts were made to compare the Ut isoforms of *P. annectens* with *H. sapiens* UT-B *and Desulfovibrio vulgaris* dvUT, as their protein structure had been used to formulate the theories of urea transport (Levin et al., 2009, 2012). An alignment of Ut-a2a and Ut-a2b from *P. annectens* with those from human, mouse, frog, three fishes (coelacanth, dogfish shark, and gulf toadfish) and bacteria revealed highly conserved LPXXTXPF motifs and the lack of UT-B ALE domain (Figure 1). In Ut-a2a of *P. annectens*, 8 out of 14 urea binding sites were conserved as compared to *D. vulgaris* dvUT (Figure 1). The urea binding sites in *D. vulgaris* dvUT, F197, L249, E307, F310, F363, and L413, were replaced with Y197, F249, Q307, G310, Y363, and C413 in Ut-a2a of *P. annectens* (Figure 1). In Ut-a2b of *P. annectens*, 9 out of 14 urea binding sites were conserved as compared to *D. vulgaris* dvUT (Figure 1). The urea binding sites of *D. vulgaris* dvUT, F197, E307, F310, F363, and L413, were replaced with Y197, Q307, G310, Y363, and F413 in Ut-a2b of *P. annectens* (Figure 1). There were 1 and 2 potential *N*-glycosylation site(s) in Ut-a2a and Ut-a2b of *P. annectens*, respectively (Figure 1). Furthermore, there were 10 and 25 potential phosphorylation sites in Ut-a2a and Ut-a2b of *P. annectens*, respectively (Figure 1). Comparison of the deduced amino acid sequences of the two Ut isoforms of *P. annectens* with Ut/UT isoforms of other animal species (Tables S2, S3) confirmed that Ut-a2a and Ut-a2b of *P. annectens* belonged to the Ut-a2/UT-A2 group. Furthermore, Ut-a2a and Ut-a2b of *P. annectens* had closer phylogenetic relationships with tetrapod Ut/UT than fish Ut (Figure 2).

### mRNA expression of *ut-a2a* and *ut-a2b* in various tissues/organs of *P. annectens*

**Figure 3:**
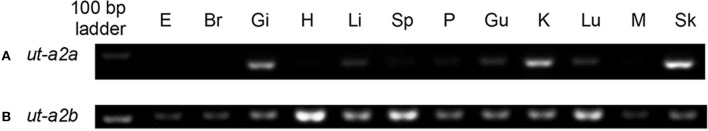
**Gene expression of (A)** *urea transporter a2a* (*ut-a2a*) and **(B)** *urea transporter a2b* (*ut-a2b*) in the eye (E), brain (Br), gills (Gi), heart (H), liver (Li), spleen (Sp), pancreas (P), gut (Gu), kidney (K), Lung (Lu), muscle (M), and skin (Sk) of *P. annectens* kept in fresh water.

For *P. annectens* kept in fresh water, *ut-a2a* was expressed strongly in the gills, kidney and skin, but weakly in the liver, gut and lung (Figure 3A). In comparison, there were strong expression of *ut-a2b* in the heart, spleen and lung, but weak expression of *ut-a2b* in the eyes, brain, gills, liver, pancreas, gut, kidney, muscle, and skin (Figure 3B).

### Branchial mRNA expression levels of *ut-a2a* and *ut-a2b* in *P. annectens* during the three phases of aestivation

**Figure 4:**
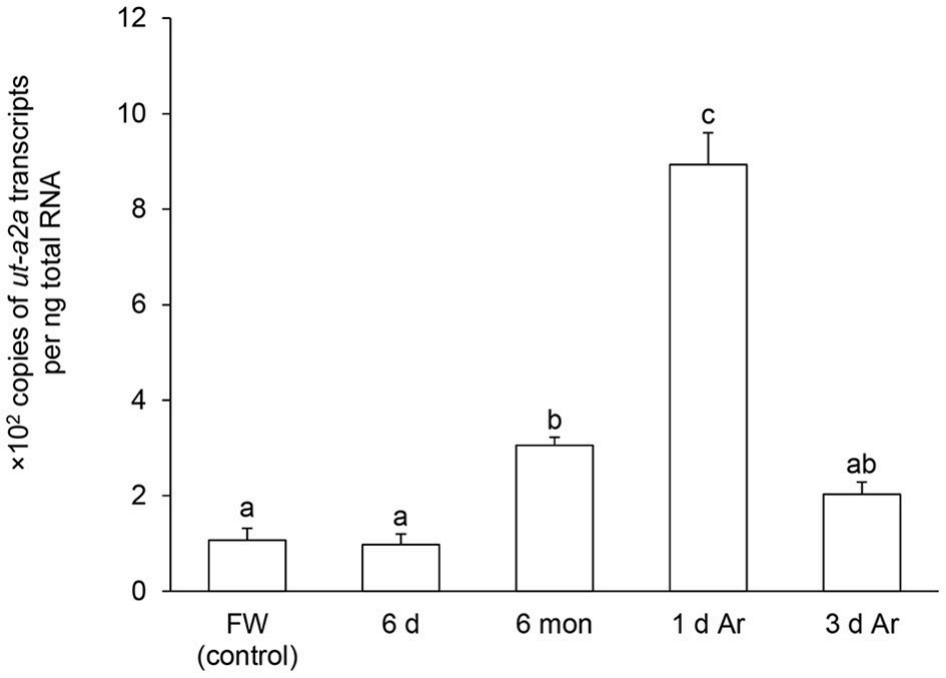
**Absolute quantification of mRNA (×10^2^ copies of transcripts per ng total RNA) of *urea transporter a2a (ut-a2a)* in the gills of *Protopterus annectens* kept in fresh water on day 0 (FW; control), after 6 days (d; induction phase), or 6 months (mon; maintenance phase) of aestivation, or after 1 or 3 d of arousal (Ar) from 6 mon of aestivation**. Results represent means ± *S.E.M* (*N* = 4). Means not sharing the same letter are significantly different (*P* < 0.05).

**Figure 5:**
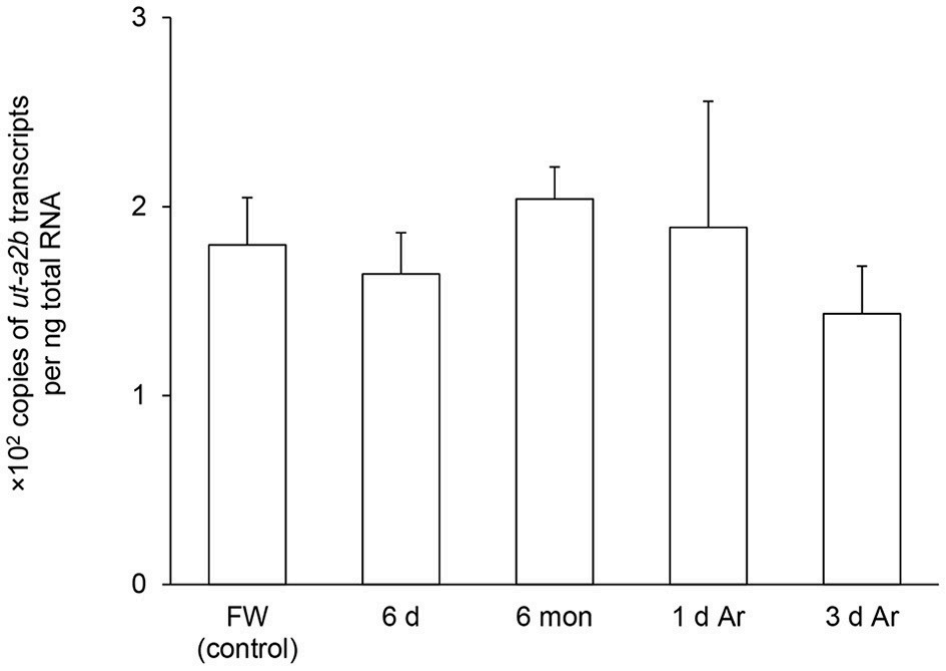
**Absolute quantification of mRNA (×10^2^ copies of transcripts per ng total RNA) of *urea transporter a2b* (*ut-a2b*) in the gills of *Protopterus annectens* kept in fresh water on day 0 (FW; control), after 6 days (d; induction phase) or 6 months (mon; maintenance phase) of aestivation, or after 1 or 3 d of arousal (Ar) from 6 mon of aestivation**. Results represent means ± *S.E.M* (*N* = 4).

After 6 days of aestivation, the mRNA expression level of *ut-a2a* in the gills of *P. annectens* remained comparable to those of control fish (Figure 4). Nevertheless, there were significant increases in the mRNA expression level of *ut-a2a* after 6 months (3.1-fold; *P* < 0.05) of aestivation or after 1 day (8.5-fold; *P* < 0.05) of arousal from 6 months of aestivation, as compared to control fish (Figure 4). The mRNA expression level of *ut-a2a* returned to control level after 3 days of arousal from 6 months of aestivation (Figure 4). No significant changes in the mRNA expression levels of *ut-a2b* were observed in the gills of *P. annectens* during the three phases of aestivation (Figure 5).

### Branchial protein abundance of Ut-a2a and Ut-a2b in *P. annectens* during the three phases of aestivation

**Figure 6:**
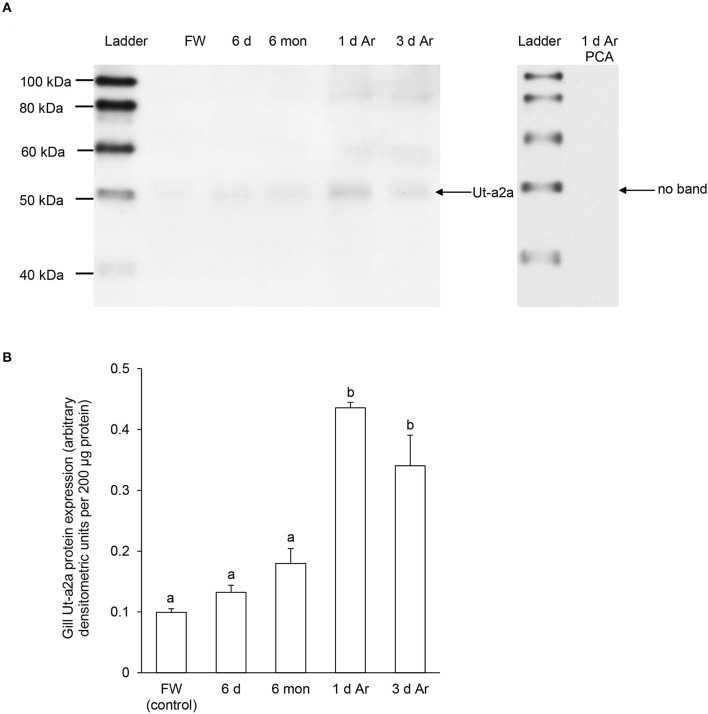
**Protein abundance of urea transporter a2a (Ut-a2a) in the gills of *Protopterus annectens* kept in fresh water on day 0 (FW; control), after 6 days (d; induction phase) or 6 months (mon; maintenance phase) of aestivation, or after 1 or 3 d of arousal (Ar) from 6 mon of aestivation**. **(A)** An example of immunoblot of Ut-a2a (left) and Ut-a2a pre-incubated with immunising peptide for the peptide competition assay (PCA; right). **(B)** The protein abundance of Ut-a2a expressed as arbitrary densitometric units per 200 μg protein. Results represent mean ± *S.E.M*. (*N* = 3). Means not sharing the same letter are significantly different (*P* < 0.05).

A band of ~50 kDa that was close to the expected molecular mass of Ut-a2a of *P. annectens* was detected using the custom-made anti-Ut-a2a antibody and a peptide competition test confirmed the validity of antibody binding (Figure 6A). After 6 days or 6 months of aestivation, the branchial protein abundance of Ut-a2a remained comparable to the control (Figure 6B). However, after 1 day and 3 days of arousal from 6 months of aestivation, it increased significantly by 4.4-fold (*P* < 0.05) and 3.4-fold (*P* < 0.05), respectively, as compared to the control (Figure 6B).

**Figure 7:**
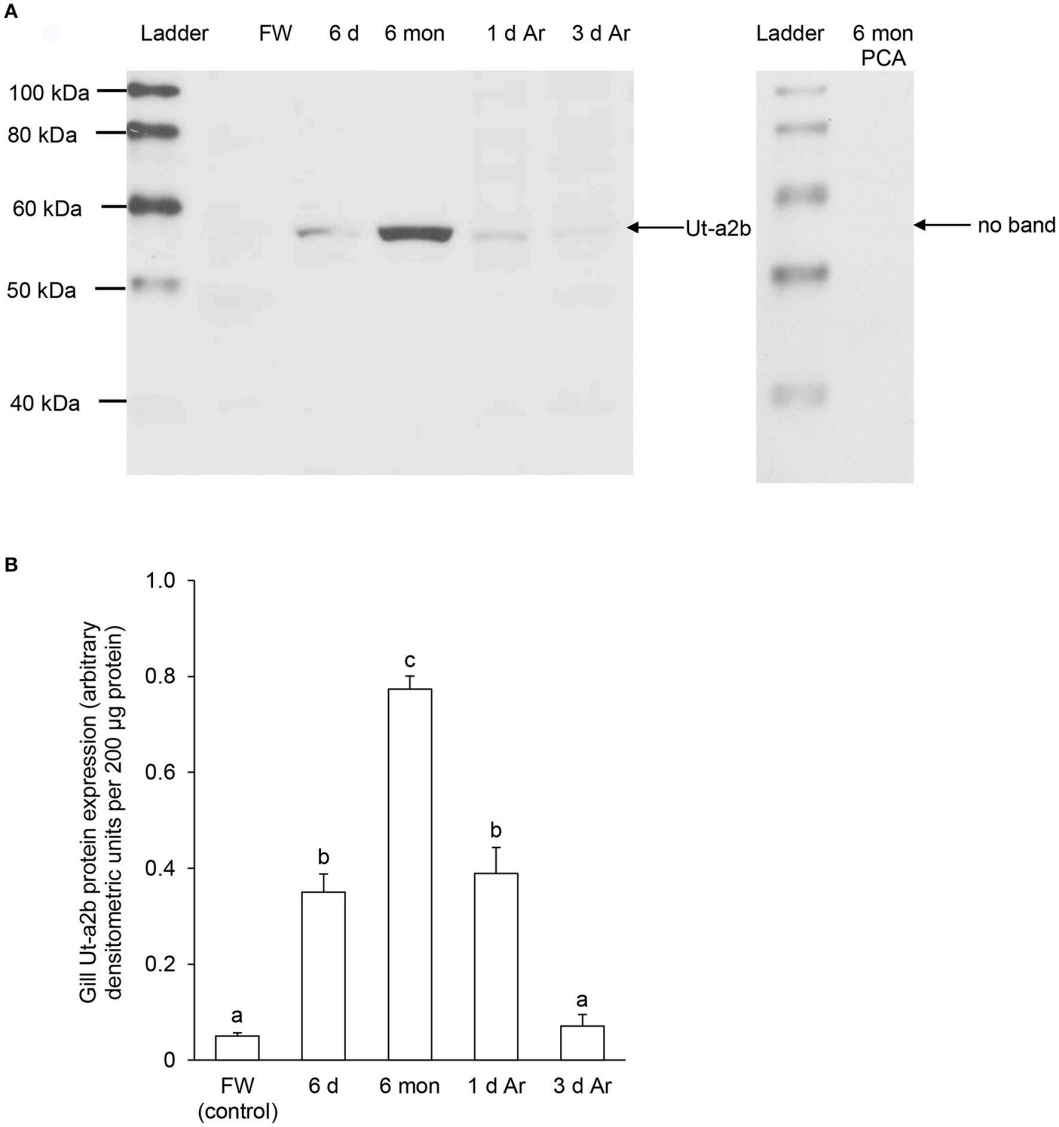
**Protein abundance of urea transporter a2b (Ut-a2b) in the gills of *Protopterus annectens* kept in fresh water on day 0 (FW; control), after 6 days (d; induction phase) or 6 months (mon; maintenance phase) of aestivation, or after 1 or 3 d of arousal (Ar) from 6 mon of aestivation**. **(A)** An example of immunoblot of Ut-a2b (left) and Ut-a2b pre-incubated with immunising peptide for the peptide competition assay (PCA; right). **(B)** The protein abundance of Ut-a2b expressed as arbitrary densitometric units per 200 μg protein. Results represent mean ± *S.E.M*. (*N* = 3). Means not sharing the same letter are significantly different (*P* < 0.05).

For Ut-a2b, a band at ~55 kDa that was close to the expected molecular mass of Ut-a2b of *P. annectens* was detected using the custom-made anti-Ut-a2a antibody and a peptide competition test confirmed the validity of antibody binding (Figure 7A). Significant increases in the protein abundance of Ut-a2b were observed in the gills of *P. annectens* after 6 days (7.0-fold; *P* < 0.05) or 6 months (15.5-fold; *P* < 0.05) of aestivation, or after 1 day (7.8-fold; *P* < 0.05) of arousal from 6 months of aestivation (Figure 7B). After 3 days of arousal from 6 months of aestivation, the protein abundance of Ut-a2b in the gills of *P. annectens* returned to the control level (Figure 7B).

## Discussion

### Molecular characterization of Ut-a2a and Ut-a2b of *P. annectens*

Ut-a2a and Ut-a2b of *P. annectens* share the highest sequence similarity with Ut-a2/UT-A2 of other animals and lack the ALE motif present in UT-B, confirming that they belong to the Ut-a2/UT-A2 group. The presence of 10 transmembrane domains in the two Ut isoforms of *P. annectens* are in agreement with the typical topology found in bacterial and mammalian Ut/UT models (You et al., 1993; Levin et al., 2009; Raunser et al., 2009), alluding to their possible function in urea transport.

Using Monte Carlo methods, molecular dynamics simulations and the adaptive biasing force approach, potential urea binding sites (Q24, V25, F27, F80, L84, L129, T130, E187, V188, F190, F243, L247, L293, and T294) have been identified in dvUT of *D. vulgaris* (Wang et al., 2015). Q24, V25, F27, L84, T130, V188, L247, and T294 of dvUT are conserved in Ut-a2a and Ut-a2b of *P. annectens*. F80, F190, and F243 play important roles in the transport selectivity of dvUT by differentiating the shape, size, and electronic configuration of potential substrates (Shayakul et al., 2013). Furthermore, these phenylalanine residues determine the orientation of the urea substrate for transport (Shayakul et al., 2013). Therefore, the replacement of F80, F190, and F243 in dvUT with tyrosine, glycine and tyrosine, respectively, in Ut-a2a and Ut-a2b of *P. annectens* could potentially alter the shape of urea pore and transport selectivity. Based on the structures of dvUT, Q24, V25, E187 and V188 form two linear arrays of three oxygen atoms, which can establish hydrogen bonding with the amide hydrogen atoms of the urea molecule (Wang et al., 2015). On the other hand, L129, T130, L293 and T294 provide amide hydrogens or hydroxyls that can potentially form hydrogen bonds with the carbonyl oxygen atoms of urea (Wang et al., 2015). L129 is conserved in Ut-a2b, but is replaced with phenylalanine in Ut-a2a of *P. annectens*. E187 is replaced with leucine in Ut-a2a and glutamine in Ut-a2b, while L293 is replaced with cysteine in Ut-a2a and phenylalanine in Ut-a2b of *P. annectens*. Since L129, E187 and L293 contribute to the formation of hydrogen bonds with urea, the replacement of these residues could potentially affect urea binding in the respective Ut isoforms of *P. annectens*.

The deduced amino acid sequences of Ut-a2a and Ut-a2b of *P. annectens* contain several potential phosphorylation sites and at least one *N*-glycosylation site, which are in agreement with those of UT-A and UT-B of *H. sapiens* (You et al., 1993; Olives et al., 1994). The presence of the phosphorylation and glycosylation sites in Ut-a2a and Ut-a2b of *P. annectens* implies that they can be regulated through post-translational modifications in response to changes in environmental conditions. Furthermore, this could explain why Ut-a2a (with an estimated molecular mass of ~44.7 kDa) and Ut-a2b (with an estimated molecular mass of ~51.2 kDa) would exhibit a higher molecular mass of ~50 and ~55 kDa, respectively, through Western blotting. Similarly, it has been reported that the bands of Ut/UT of *Rhinella marina* (Konno et al., 2006), *Triakis scyllium* (Hyodo et al., 2004), and *Homo sapiens* (Wade et al., 2000), despite having estimated molecular masses ranging from 42.7 to 43.4 kDa, display higher molecular masses (~50 to ~55 kDa) on immunoblots. It has been postulated previously that post-translational modifications of Ut/UT are likely to account for the higher molecular mass detected through immunoblotting (Wade et al., 2000; Bradford et al., 2001).

### Ut-a2a and Ut-a2b of *P. annectens* are grouped with those of tetrapods

Based on several molecular phylogenetic studies (Takezaki et al., 2004; Hallström and Janke, 2009; Amemiya et al., 2013), lungfishes are believed to be the closest living relatives of tetrapods. Indeed, our dendrogramic analysis indicated that Ut-a2a and Ut-a2b are grouped closer to tetrapods. This result is in agreement with the dendrogramic grouping of some proteins of *P. annectens*, which include Asl (Chng et al., 2014), Na^+^/K^+^-ATPase α-subunit isoforms (Hiong et al., 2014), Gulonolactone oxidase (Ching et al., 2014), Coagulation factor II (F2), Fibrinogen gamma chain (Hiong et al., 2015), Betaine-homocysteine *S*-methyltransferase 1 (Bhmt1; Ong et al., 2015), and Aquaporin 1 and 3 (Chng et al., 2016).

Several gene duplication events of *ut* occurred during the evolution of lower vertebrates (LeMoine and Walsh, 2015). As Hung et al. (2009) identified only one *ut* from the skin of *P. annectens*, LeMoine and Walsh (2015) postulated that the loss of duplicated *ut* isoforms could have occurred prior to the Sarcopterygian radiation. Furthermore, the presence of a single homologous *ut* gene in amphibians supports the early pseudogenization or deletion of duplicated *ut* genes in primitive tetrapod lineages (LeMoine and Walsh, 2015). As two *ut*/Ut isoforms are expressed in *P. annectens*, it is logical to deduce that the loss of duplication of *ut* isoforms could not have occurred prior to or during the Sarcopterygian radiation, but transpired during the evolution of amphibians.

### *ut-a2a* and *ut-a2b* have comparable transcript levels in the gills of *P. annectens*

The expression of *ut-a2a* in the skin was much stronger than that in the gills of control fish kept in fresh water. In the skin, *ut-a2a* was the major *ut* isoform as *ut-a2b* was only weakly expressed. This could be the reason why Hung et al. (2009) reported only the *ut-a2a*-equivalent (*lfut*) from the skin of *P. annectens*. As for the gills, the expression levels of *ut-a2a* and *ut-a2b* appeared to be comparable, although quantitative conclusions cannot be drawn based on PCR results alone. Using qPCR, we confirmed that the gills of *P. annectens* kept in fresh water had similar transcript levels of *ut-a2a* and *ut-a2b*. Therefore, it is logical to deduce that both Ut-a2a and Ut-a2b would contribute to urea excretion through the gills of *P. annectens*, particularly during the arousal phase. More importantly, the transcript levels and protein abundance of *ut-a2a*/Ut-a2a and *ut-a2b*/Ut-a2b remained either unchanged or increased significantly in the gills during the maintenance phase, which were unexpected as no urea excretion could have occurred.

### Changes in branchial expression of *ut-a2a*/Ut-a2a in *P. annectens* during the three phases of aestivation

There were no changes in the transcript level and protein abundance of *ut-a2a*/Ut-a2a in the gills of *P. annectens* after 6 days (the induction phase) of aestivation. However, the transcript level of *ut-a2a* increased significantly while the protein abundance of Ut-a2a remained unchanged as compared with the control after 6 months of aestivation (the maintenance phase). The significant increase in *ut-a2a* transcripts cannot be explained by decreased degradation of *ut-a2a* transcripts alone. There must be continuous transcription of *ut-a2a* in the gills of *P. annectens* during the maintenance phase of aestivation. It is apparent that the transcriptional *ut-a2a* and translation of Ut-a2a is momentarily disengaged resulting in the unchanged protein abundance of Ut-a2a in the gills. This can be interpreted as an adaptive feature to prepare for arousal, as the increased transcript level of *ut-a2a* would prepare for a subsequent increase in the production of Ut-a2a during the arousal phase. As expected, the *ut-a2a* transcripts in the gills continued to increase upon arousal (1 day) from 6 months of aestivation and the Ut-a2a protein abundance increased concurrently by ~4-fold. These results confirm that the increase in translation of Ut-a2a is activated when water becomes available upon arousal. In addition, they imply that, besides the skin (Hung et al., 2009), the gills also contribute to facilitating the excretion of urea accumulated in the body immediately upon arousal.

### Changes in branchial expression of *ut-a2b*/Ut-a2b in *P. annectens* during the three phases of aestivation

In contrast with *ut-a2a*/Ut-a2a, *ut-a2b*/Ut-a2b was regulated mainly at the translational level. While the transcript level of *ut-a2b* remained unchanged throughout the three phases of aestivation, there were significant increases in Ut-a2b protein abundance in the gills of *P. annectens* during the induction phase (7.0-fold), the maintenance phase (15.5-fold), and the arousal phase (7.8-fold) as compared with the control. While a decrease in the degradation of Ut-a2b might have contributed in part to the continuous increase of Ut-a2b protein abundance during the 6 months of aestivation, there must be an up-regulation in the translation of Ut-a2b between the induction phase and the maintenance phase of aestivation. Of note, during the induction and maintenance phases, *P. annectens* gills are covered with a thick layer of mucus (Sturla et al., 2001), and branchial functions such as respiration, osmoregulation and nitrogenous waste excretion should come to a halt. Theoretically, the gills should undergo molecular changes to shut down branchial functions. As down-regulation of gene and protein expression would save metabolic energy and conserve endogenous metabolic fuel, the expression of unimportant transporters is expected to be suppressed during the maintenance phase of aestivation. Since that is not the case for Ut-a2b, it would imply that the up-regulation of translation of Ut-a2b represents an adaptive mechanism to survive the aestivation process.

Urea can act as an osmolyte to maintain cell volume in conditions of osmotic stress (Yancey, 2005; LeMoine and Walsh, 2015). Some frogs, such as *Scaphiopus couchii*, accumulate urea during aestivation (Jones, 1980). The high concentration of urea could promote favorable osmotic gradient for water transfer between the frog and its environment, suggesting that urea could function as a balancing osmolyte (Jones, 1980). It is possible that increase in Ut-a2b protein abundance could facilitate urea accumulation for cell volume regulation in aestivating lungfish. Furthermore, an important facet of aestivation is that the lungfish must be able to arouse from aestivation upon re-immersion; if not, it must have somehow succumbed to the aestivation process during the maintenance phase. Hence, making preparations for successful arousal is essential, and the increase in Ut-a2b protein abundance may be regarded as an adaptive response to get ready for the efficient excretion of the accumulated urea when water becomes available. As there are indications that the accumulated urea can act as the internal cue for aestivation in African lungfishes (Ip et al., 2005a), reversibly inhibit key metabolic enzymes (Hand and Somero, 1982; Yancey et al., 1982), and contribute to metabolic depression in dormant animals (Griffith, 1991) like the hibernating wood frog, *Rana sylvatica* (Muir et al., 2008), it is obviously important for *P. annectens* to eliminate it from the body with high efficiency during the initial phase of arousal. Similarly, marsupials (Hume, 1982) and Columbian ground squirrel (Passmore et al., 1975) accumulate urea during hibernation and excrete it promptly upon arousal.

### Insights into transcriptional and translational processes in *P. annectens* during the maintenance phase of aestivation

As conservation of metabolic fuels is essential during extended periods of aestivation without food, metabolic depression has long been regarded as an important feature of aestivation (Storey, 2002). Moreover, as transcription and translation are energy-intensive processes, the assumption is that strong global suppression of gene expression and protein synthesis should occur during metabolic depression (Storey and Storey, 2010). Nevertheless, it should not be generalized that all transcriptional and translational activities are suppressed in every organs during the three phases of aestivation. Results from this study indicated that there were significant increases in the mRNA expression level of *ut-a2a* and the protein abundance of Ut-a2b in the gills of *P. annectens* after 6 months of aestivation. Similarly, the mRNA expression levels of *cps III* (Loong et al., 2012), *ass, asl* (Chng et al., 2014), and *bhmt1* (Ong et al., 2015) in the liver, and that of *asl* in the brain (Chng et al., 2014), increased significantly in *P. annectens* during the maintenance phase of aestivation. In addition, increases in protein abundance during the maintenance phase have been reported for Bhmt1 in the liver and muscle (Ong et al., 2015), F2 in the liver (Hiong et al., 2015), hypoxia inducible factor 1α in the gills (Garofalo et al., 2015), and activated eNOS/Akt and heat shock protein 90 in the lung (Garofalo et al., 2015) of *P. annectens*. Taken together, these results indicate that the supposition of expression and syntheses of all genes/proteins being suppressed in every organ in an African lungfish during the maintenance phase of aestivation may not be valid (Ip and Chew, 2010a; Chew et al., 2015). They support the notion that aestivation cannot be viewed simply as the result of a general metabolic depression, but it encompasses the complex interplay between down-regulation and up-regulation of many cellular processes.

## Author contributions

YI designed the experiment. JO, BC, XC, KH, WW, and SC were involved in the subjection of animal and the collection of samples. YC performed all experiments and analyzed the data. SL and YI were involved in the analysis of data and the approval of manuscript. YI and YC wrote the manuscript. All authors were involved in the revision of the manuscript.

### Conflict of interest statement

The authors declare that the research was conducted in the absence of any commercial or financial relationships that could be construed as a potential conflict of interest.

## Note on abbreviations

As there are differences in the abbreviations used for genes/proteins of fish (http://wiki.zfin.org/display/general/ZFIN+Zebrafish+Nomenclature+Guidelines) and human/non-human primates (http://www.genenames.org), we adopted two different types of abbreviations in this report. The symbols for fish genes are italicized and in lower case, while the protein designations are similar to the gene symbols, but not italicized with the first letter in upper case.
