# Supplementary material for: Aestivation Induces Changes in the mRNA Expression Levels and Protein Abundance of Two Isoforms of Urea Transporters in the Gills of the African Lungfish, Protopterus annectens
Source: Front Physiol. 2017 Feb 16;8:71. doi: 10.3389/fphys.2017.00071 (PMC5311045; doi:10.3389/fphys.2017.00071)
Supplement: Supplementary file 1 [file Table1.DOCX]

Table S1. List of selected species and their accession numbers used for dendrogram analysis of Ut/UT. “*” indicates the outgroup.

| Species | Accession number |
| --- | --- |
| *Alcolapia grahami* Ut-a2 | AAG49891.1 |
| *Anguilla japonica* Ut-a2 | BAC53976.1 |
| *Anguilla japonica* Ut-c | BAD66672.1 |
| *Bos taurus* UT-A2 | NP_001008666.1 |
| *Bos taurus* UT-B | NP_001137574.1 |
| *Bos taurus* UT-B isoform x2 | XP_005224169.1 |
| *Callorhinchus milii* Ut-a2a | BAH58773.1 |
| *Callorhinchus milii* Ut-a2b | BAH58774.1 |
| *Callorhinchus milii* Ut-c | BAH58777.1 |
| *Callorhinchus milii* UT-da | BAH58775.1 |
| *Callorhinchus milii* Ut-db | BAH58776.1 |
| *Cynoglossus semilaevis* Ut-a2 | XP_008335055.1 |
| *Danio rerio* Ut-a2 | NP_001018355.1 |
| *Dasyatis sabina* Ut-a2a | AAQ07592.1 |
| *Dasyatis sabina* Ut-a2b | AAQ23380.1 |
| *Dasyatis sabina* Ut-a2c | AAQ23381.1 |
| *Dasyatis sabina* Ut-da | AAQ23379.1 |
| *Dasyatis sabina* Ut-db | AAM46683.2 |
| *Dasyatis say* Ut-a2 | AAQ23382.1 |
| *Esox lucius* Ut-a2 | XP_010874746.1 |
| *Homo sapiens* UT-A1 | AAL08485.1 |
| *Homo sapiens* UT-A2 | CAA65657.1 |
| *Homo sapiens* UT-B1 | CAB60834.1 |
| *Larimichthys crocea* Ut-a2 | KKF10186.1 |
| *Larimichthys crocea* Ut-d isoform x1 | XP_010731977.1 |
| *Larimichthys crocea* Ut-d isoform x2 | KKF21937.1 |
| *Latimeria chalumnae* Ut-a2 | XP_006007026.1 |
| *Leucoraja ocellata* Ut-a2 | AAL12243.1 |
| *Mus musculus* UT-A1 | AAM00357.1 |
| *Mus musculus* UT-A2 | AAM21206.1 |
| *Mus musculus* UT-A3 | AAG32168.1 |
| *Mus musculus* UT-A5 | AAG32167.1 |
| *Mus musculus* UT-B | AAL47138.1 |
| *Opsanus beta* Ut-a2 | AAD53268.2 |
| *Oryzias latipes* Ut-a2 | XP_004072672.2 |

Table S1 (continued)

| Species | Accession number |
| --- | --- |
| *Pelophylax esculentus* Ut-a2 | CAA73322.1 |
| *Poecilia formosa* Ut-a2 | XP_007559324.1 |
| *Porichthys notatus* Ut-a2 | AGA93882.1 |
| *Rana pipiens* Ut-a2 | AFE48182.1 |
| *Rana septentrionalis* Ut-a2 | AFE48183.1 |
| *Rana sylvatica* Ut-a2 | AFE48181.1 |
| *Rattus norvegicus* UT-A1 | AAB50197.1 |
| *Rattus norvegicus* UT-A2 | AAB39937.1 |
| *Rattus norvegicus* UT-A3 | AAD23098.1 |
| *Rattus norvegicus* UT-A4 | AAD23099.1 |
| *Rattus norvegicus* UT-B | NP_062219.2 |
| *Rhinella marina* Ut-a2 | BAE16706.1 |
| *Scyliorhinus canicula* Ut-a2 | AEH59797.1 |
| *Squalus acanthias* Ut-a2 | AAF66072.1 |
| *Takifugu rubripes* Ut-a2 | BAD66674.1 |
| *Takifugu rubripes* Ut-c | NP_001033079.1 |
| *Triakis scyllium* Ut-a2 | BAC75980.1 |
| *Xiphophorus maculatus* Ut-a2 | XP_005804110.1 |
| *Strongylocentrotus purpuratus* Ut* | XP_786452.3 |
