# Supplementary material for: Aestivation Induces Changes in the mRNA Expression Levels and Protein Abundance of Two Isoforms of Urea Transporters in the Gills of the African Lungfish, Protopterus annectens
Source: Front Physiol. 2017 Feb 16;8:71. doi: 10.3389/fphys.2017.00071 (PMC5311045; doi:10.3389/fphys.2017.00071)
Supplement: Supplementary file 2 [file Table2.DOCX]

Table S2. The percentage similarity between the deduced amino acid sequence of urea transporter A2a (Ut-a2a) from *Protopterus annectens* and other Ut/UT sequences from other animal species obtained from GenBank (accession numbers in brackets). Sequences are arranged in a descending order of similarity.

| Classification | Species | Similarity |
| --- | --- | --- |
| Sarcopterygians | *Protopterus annectens* Ut-a2b | 71.3% |
|  | *Latimeria chalumnae* Ut-a2 (XP_006007026.1) | 60.1% |
| Amphibians | *Rhinella marina* Ut-a2 (BAE16706.1) | 67.4% |
|  | *Pelophylax esculentus* Ut-a2 (CAA73322.1) | 63.2% |
|  | *Rana sylvatica* Ut-a2 (AFE48181.1) | 62.5% |
|  | *Rana septentrionalis* Ut-a2 (AFE48183.1) | 62.2% |
|  | *Rana pipiens* Ut-a2 (AFE48182.1) | 62.2% |
| Chondrichthyes | *Squalus acanthias* Ut-a2 (AAF66072.1) | 62.1% |
|  | *Leucoraja ocellata* Ut-a2 (AAL12243.1) | 61.1% |
|  | *Triakis scyllium* Ut-a2 (BAC75980.1) | 60.0% |
|  | *Callorhinchus milii* Ut-a2b (BAH58774.1) | 59.5% |
|  | *Dasyatis sabina* Ut-a2a (AAQ07592.1) | 58.7% |
|  | *Dasyatis sabina* Ut-a2b (AAQ23380.1) | 58.7% |
|  | *Dasyatis sabina* Ut-a2c (AAQ23381.1) | 58.7% |
|  | *Scyliorhinus canicula* Ut-a2 (AEH59797.1) | 58.3% |
|  | *Dasyatis say* Ut-a2 (AAQ23382.1) | 58.0% |
|  | *Callorhinchus milii* Ut-a2a (BAH58773.1) | 56.8% |
|  | *Dasyatis sabina* Ut-da (AAQ23379.1) | 52.6% |
|  | *Dasyatis sabina* Ut-db (AAM46683.2) | 52.6% |
|  | *Callorhinchus milii* Ut-db (BAH58776.1) | 52.2% |
|  | *Callorhinchus milii* UT-da (BAH58775.1) | 47.9% |
|  | *Callorhinchus milii* Ut-c (BAH58777.1) | 43.8% |
| Mammals | *Homo sapiens* UT-A2 (CAA65657.1) | 60.4% |
|  | *Mus musculus* UT-A2 (AAM21206.1) | 59.7% |
|  | *Mus musculus* UT-B (AAL47138.1) | 55.8% |
|  | *Rattus norvegicus* UT-B (NP_062219.2) | 55.2% |
|  | *Rattus norvegicus* UT-A2 (AAB39937.1) | 54.9% |
|  | *Homo sapiens* UT-B1 (CAB60834.1) | 54.6% |
|  | *Bos taurus* UT-A2 (NP_001008666.1) | 53.1% |
|  | *Rattus norvegicus* UT-A4 (AAD23099.1) | 52.2% |
|  | *Mus musculus* UT-A5 (AAG32167.1) | 51.8% |
|  | *Mus musculus* UT-A3 (AAG32168.1) | 50.4% |
|  | *Rattus norvegicus* UT-A3 (AAD23098.1) | 50.1% |
|  | *Bos taurus* UT-B isoform x2 (XP_005224169.1) | 46.9% |

Table S2 (continued)

| Classification | Species | Similarity |
| --- | --- | --- |
| Mammals | *Bos taurus* UT-B (NP_001137574.1) | 27.7% |
|  | *Homo sapiens* UT-A1 (AAL08485.1) | 27.5% |
|  | *Mus musculus* UT-A1 (AAM00357.1) | 25.1% |
|  | *Rattus norvegicus* UT-A1 (AAB50197.1) | 25.0% |
| Actinopterygians | *Porichthys notatus* Ut-a2 (AGA93882.1) | 50.2% |
|  | *Danio rerio* Ut-a2 (NP_001018355.1) | 49.1% |
|  | *Larimichthys crocea* Ut-a2 (KKF10186.1) | 49.0% |
|  | *Xiphophorus maculatus* Ut-a2 (XP_005804110.1) | 49.0% |
|  | *Anguilla japonica* Ut-a2 (BAC53976.1) | 48.6% |
|  | *Alcolapia grahami* Ut-a2 (AAG49891.1) | 48.2% |
|  | *Poecilia formosa* Ut-a2 (XP_007559324.1) | 47.5% |
|  | *Opsanus beta* Ut-a2 (AAD53268.2) | 47.2% |
|  | *Esox lucius* Ut-a2 (XP_010874746.1) | 47.1% |
|  | *Takifugu rubripes* Ut-a2 (BAD66674.1) | 47.0% |
|  | *Oryzias latipes* Ut-a2 (XP_004072672.2) | 45.8% |
|  | *Larimichthys crocea* Ut-d isoform x1 (XP_010731977.1) | 45.5% |
|  | *Cynoglossus semilaevis* Ut-a2 (XP_008335055.1) | 45.5% |
|  | *Larimichthys crocea* Ut-d isoform x2 (KKF21937.1) | 34.4% |
|  | *Anguilla japonica* Ut-c (BAD66672.1) | 34.2% |
|  | *Takifugu rubripes* Ut-c (NP_001033079.1) | 30.3% |
